# Intermediate-Intensity Autologous Hematopoietic Stem Cell Transplantation Reduces Serum Neurofilament Light Chains and Brain Atrophy in Aggressive Multiple Sclerosis

**DOI:** 10.3389/fneur.2022.820256

**Published:** 2022-02-24

**Authors:** Alice Mariottini, Leonardo Marchi, Chiara Innocenti, Maria Di Cristinzi, Matteo Pasca, Stefano Filippini, Alessandro Barilaro, Claudia Mechi, Arianna Fani, Benedetta Mazzanti, Tiziana Biagioli, Francesca Materozzi, Riccardo Saccardi, Luca Massacesi, Anna Maria Repice

**Affiliations:** ^1^Department of Neurosciences, Drug and Child Health, University of Florence, Florence, Italy; ^2^Department of Neurology 2 and Tuscan Region Multiple Sclerosis Referral Centre, Careggi University Hospital, Florence, Italy; ^3^Cell Therapy and Transfusion Medicine Unit, Careggi University Hospital, Florence, Italy; ^4^General Laboratory, Careggi University Hospital, Florence, Italy

**Keywords:** hematopoietic (stem) cell transplantation (HSCT), multiple sclerosis, neurofilament light (NfL), biomarker, brain atrophy, PIRA, progression independent of relapse activity

## Abstract

**Background:**

Autologous haematopoietic stem cell transplantation (AHSCT) is highly effective in reducing new inflammatory activity in aggressive multiple sclerosis (MS). A remarkable decrease of serum neurofilament light chains (sNfL) concentration, a marker of axonal damage, was reported in MS following high-intensity regimen AHSCT, but hints for potential neurotoxicity had emerged. sNfL and brain atrophy were therefore analysed in a cohort of patients with aggressive MS treated with intermediate-intensity AHSCT, exploring whether sNfL might be a reliable marker of disability progression independent from new inflammation (i.e. relapses and/or new/gadolinium-enhancing MRI focal lesions).

**Methods:**

sNfL concentrations were measured using SIMOA methodology in peripheral blood from relapsing-remitting (RR-) or secondary-progressive (SP-) MS patients undergoing AHSCT (MS AHSCT), collected before transplant and at months 6 and 24 following the procedure. sNfL measured at a single timepoint in SP-MS patients not treated with AHSCT without recent inflammatory activity (SP-MS CTRL) and healthy subjects (HD) were used as controls. The rate of brain volume loss (AR-BVL) was also evaluated by MRI in MS AHSCT cases.

**Results:**

Thirty-eight MS AHSCT (28 RR-MS; 10 SP-MS), 22 SP-MS CTRL and 19 HD were included. Baseline median sNfL concentrations were remarkably higher in the MS AHSCT than in the SP-MS CTRL and HD groups (*p* = 0.005 and <0.0001, respectively), and levels correlated with recent inflammatory activity. After a marginal (not significant) median increase observed at month 6, at month 24 following AHSCT sNfL concentrations decreased compared to baseline by median 42.8 pg/mL (range 2.4–217.3; *p* = 0.039), reducing by at least 50% in 13 cases, and did not differ from SP-MS CTRL (*p* = 0.110) but were still higher than in HD (*p* < 0.0001). Post-AHSCT AR-BVL normalised in 55% of RR-MS and in 30% of SP-MS. The effectiveness and safety of AHSCT were aligned with the literature.

**Conclusion:**

sNfL concentrations correlated with recent inflammatory activity and were massively and persistently reduced by intermediate-intensity AHSCT. Association with response to treatment assessed by clinical or MRI outcomes was not observed, suggesting a good sensitivity of sNfL for recent inflammatory activity but low sensitivity in detecting ongoing axonal damage independent from new focal inflammation.

## Introduction

Autologous haematopoietic stem cell transplantation (AHSCT) is a treatment option for a selected population of patients with aggressive multiple sclerosis (aMS), endorsed as “standard of care” for treatment-refractory relapsing MS (1, 2). AHSCT virtually eradicates new inflammatory activity in MS, and its risk-benefit ratio is highly favourable in early inflammatory phases of the disease (relapsing-remitting, RR-), whereas efficacy in secondary-progressive (SP-) MS is still controversial and long-term stabilisation of disability is achieved only in a moderate proportion of cases (3).

Neurofilament light chain (NfL) concentrations, which can be reliably evaluated in cerebrospinal fluid (CSF) and serum, are associated in MS patients with inflammatory activity, disability accrual, and accelerated brain atrophy; serum NfL (sNfL) has been proposed as a useful biomarker for treatment monitoring (4–6). A remarkable decrease in NfL levels was recently reported in paired CSF and serum samples of aMS treated with high-intensity conditioning AHSCT (7), but a possible transient neurotoxic effect of this protocol was suggested by a temporary increase in NfL detected shortly after the procedure (8). Similarly, hints of potential neurotoxicity of high-intensity conditioning AHSCT emerged from other studies (9, 10), but no data are available so far on intermediate-intensity regimens AHSCT. We hence present the results of a monocentric study evaluating the effect of intermediate intensity regimen AHSCT on sNfL and brain atrophy in aMS, exploring whether in this cohort sNfL might be a reliable marker of disability progression independent from new focal inflammation, thus identifying MS patients non-responding to the procedure.

## Materials and Methods

### Patient and Control Populations

#### MS AHSCT

RR-MS or SP-MS patients diagnosed according to the Poser and Mc Donald criteria (11–13) who had been enrolled in an open-label monocentric study of AHSCT in Florence and who had frozen-stored serum samples previously collected at pre-defined timepoints (baseline, i.e. before haematopoietic stem cells mobilisation, months 6 and 24 after transplant) were included as the MS AHSCT group, according to the inclusion/exclusion criteria of the transplant centre. Briefly, RR-MS patients were considered for the procedure if they showed highly active MS despite treatment with disease-modifying treatments (DMTs) (i.e. occurrence of a disabling relapse or of at least two clinical relapses in the year prior to enrolment, associated with signs of new focal inflammatory activity at brain MRI in the previous year); or had history of highly active disease and scheduled withdrawal of a second line DMT. SP-MS were included if they had experienced a confirmed EDSS worsening in the previous year coupled with clinical or radiological evidence of new inflammatory activity in the year prior to inclusion (signs of new inflammation were not required if receiving active treatment). The main exclusion criteria were the following: primary progressive MS, pregnancy or other medical conditions that could contraindicate AHSCT, acute infections, malignancies, relevant comorbidity (e.g. liver disease, kidney failure, …), inability to provide adequate informed consent to participate to the study. Treatments were performed between 2007 and 2018 at the Cellular Therapies and Transplant Unit of the Careggi University Hospital in Florence, Italy, in collaboration with the Tuscan Region MS Referral Centre of the same hospital. Briefly, mobilisation of haematopoietic stem cells was obtained with IV cyclophosphamide (4 g/m^2^) and granulocyte colony-stimulating factor. The conditioning regimen used was BEAM+ATG, an intermediate intensity regimen according to the EBMT classification (2), for all the patients except for two individuals who received either melphalan-carmustine-ATG or BEAM without melphalan and ATG for safety issues. Standardised haematological and neurological evaluations were performed at baseline, at months 6 and 12 after transplant and then yearly. Disability was assessed as Expanded Disability Status Scale (EDSS) (14) worsening (i.e. occurrence of one single episode of EDSS deterioration, defined as an increase of at least 1.0 or 0.5 EDSS point if baseline EDSS was <5.5 or ≥5.5, respectively) and continuous disability accrual (CDA, i.e. at least two confirmed episodes of EDSS worsening associated with continuous progression of disability between timepoints), as previously reported (15). Baseline, 6-month and 24-month samples were available for 37, 33 and 37 cases, respectively. sNfL measurement at all the timepoints was available for 31 patients.

#### Control Groups

In order to explore the accuracy of sNfL concentration in detecting axonal damage independent from new focal inflammation, SP-MS patients without signs of recent inflammatory activity (relapses and/or gadolinium-enhancing—Gd+–brain lesions in the 6 months before the collection of the serum sample), of age similar to the AHSCT SP-MS cases at month 24 after treatment were included (SP-MS CTRL). In addition, people not affected by any neurological disorders (healthy controls—HD) were included as normal controls. Serum samples in these two control groups were collected at one timepoint only and frozen-stored until their utilisation.

### Laboratoristic and MRI Assessments

#### sNfL Measurement

sNfL were measured using single-molecule array (SIMOA) technology in cryopreserved serum samples stored in the same conditions; a quantification in duplicate was performed according to the manufacturer's instructions (16). Variation in absolute values within 20% was considered as not significant.

#### Magnetic Resonance Imaging Analysis

Brain magnetic resonance imaging (MRI) was performed at baseline, and then at least yearly up to the last follow-up. An additional scan at month 6 following AHSCT was available for a subset of cases.

MRI inflammatory activity was defined as the occurrence of new T2 lesions and/or Gd+ lesions in a follow-up brain MRI, compared to the baseline scan. T2 lesion load was evaluated using MIPAV software. Two-timepoint percentage brain volume change was estimated using the Structural Image Evaluation using Normalisation of Atrophy (SIENA) methodology (17, 18); whole brain volume at a timepoint (normalised for subject head size) was calculated with SIENAX, FSL-suite. The annualised rate of brain volume loss (AR-BVL) was then calculated as follows: (PBVC/100+1)^∧^(365.25/days)−1)^*^100, where PBVC is the percentage brain volume change obtained with SIENA; AR-BVL was calculated up to last available MRI. A brain volume change >-0.4%/year was considered pathological, according to normative data (19).

### Aims of the Study

The main aim of the study was to explore the impact of intermediate-intensity regimen AHSCT on sNfL in aMS patients, exploring if AHSCT could reduce sNfL to levels similar to SP-MS patients without signs of recent focal inflammation, or to healthy controls. As exploratory endpoint, potential correlations between response to AHSCT and sNfL were analysed, investigating whether in MS AHSCT treated patients sNfL could be a marker of disability accrual independent from new focal inflammation.

### Statistical Methods

Baseline characteristics of the cases are reported as median and range, or as mean and 95% CI, as appropriate. Non parametric tests were adopted to compare baseline characteristics between groups (Mann-Whitney test for continuous and Chi-square test for dichotomic variables). Correlation between sNfL concentration and the other variables were explored using partial correlation after adjusting for age at the sample. Event-free survival was estimated using the Kaplan–Meier survival analysis. A Cox regression model was adopted to explore the effect of baseline variables on the outcomes. The statistics software used were SPSS (IBM SPSS Statistics, RRID:SCR_019096) version 25 and Origin Pro for Windows. A two-tailed *p*-value < 0.05 was considered significant.

## Results

### Patient and Control Group Characteristics

Thirty-eight aMS patients (28 RR-MS and 10 SP-MS) treated with AHSCT were included (MS AHSCT). Twenty-two SP-MS patients not treated with AHSCT and 19 healthy individuals (68% females) were included in the SP-MS CTRL and HD groups, respectively. Baseline clinical and demographic characteristics of MS patients included in the study are reported in Table 1. In the AHSCT group, age at baseline was similar to that of HD (median 35 years, range 20–57, vs. median 36 years, range 26–65, respectively; *p* = 0.260); the median age in the SP-MS AHSCT group at 24 months serum collection was similar to that of the SP-MS CTRL group (median 45, range 28–59, and median 49.5, range 33–64, respectively; *p* = 0.070).

**Table 1:** Baseline clinical and demographic characteristics of the MS patients included in the study.

	**RR-MS AHSCT (*n* = 28)**		**SP-MS AHSCT (*n* = 10)**		**SP-MS CTRL (*n* = 22)**		**SP-MS AHSCT vs. SP-MS CTRL**
	**Median**	**(Range)**	**Median**	**(Range)**	**Median**	**(Range)**	***p*** **Value**
Age at baseline, y	34	(20–53)	43	(26–57)	49.5	(33–64)	0.039*
Disease duration from the onset, y	9.5	(1–22)	11	(6–23)	21.5	(6–36)	0.010*
Progressive phase duration, m	N/A	N/A	18.5	(7–79)	68	(3–181)	0.010*
Previous treatment duration with DMTs, y	6	(0–21)	7.5	(4–21)	15	(5–28)	0.006*
DMTs received, *n*	3	(0–7)	3	(2–6)	3	(1–5)	0.572
Baseline EDSS	4.0	(1.0–7.0)	5.75	(4.0–6.0)	6.25	(3.5–7.5)	0.182
Delta EDSS in the previous year	0.5	(-1.5–1.5)	0	(0–1.0)	0.25	(0–2.5)	0.257
Progression Index^a^	0.92	(0.43–1.41)	0.64	(0.34–0.94)	0.41	(0.31–0.50)	0.044*
Relapses in the previous year, *n*	1.5	(0–6)	0.5	(0–2)	0	(0–1)	0.002*
Gd+ lesions at last brain MRI, *n*	1.5	(0–31)	0.5	(0–3)	0	(0–0)	<0.001*
	**n**	**(%)**	***n***	**(%)**	***n***	**(%)**	***p*** **value**
Sex, female	22	(79%)	8	(80%)	14	(64%)	0.440
Cases with relapse in the previous 6 months	17	(61%)	3	(30%)	0	(0%)	0.024*
Cases with EDSS worsening in the previous year	6	(21%)	3	(30%)	8	(36%)	1.000
Cases receiving DMTs at blood sampling	17	(61%)	8	(80%)	13	(59%)	0.425
Cases showing Gd+ lesions in pre-treatment brain MRI	18	(64%)	5	(50%)	0	(0%)	0.001*

a *Mean (95% confidence interval—CI). N/A: not applicable*.

Significant values (p < 0.05) are marked with *.

*AHSCT, autologous haematopoietic stem cell transplantation; DMTs, disease-modifying treatments; EDSS, Expanded Disability Status Scale; Gd, gadolinium; MRI, magnetic resonance imaging; MS, multiple sclerosis; MS AHSCT, MS patients treated with AHSCT; RR-, relapsing-remitting; SP-, secondary-progressive; SP-MS CTRL, SP-MS control group (i.e. not treated with AHSCT)*.

At baseline serum collection (corresponding to pre-mobilisation for AHSCT patients, and to the single blood sample available for cases in the control groups), SP-MS AHSCT and SP-MS CTRL groups differed in the following characteristics: age (*p* = 0.039), disease duration (*p* = 0.010) and disability accrual rate (*p* = 0.044, Table 1).

At baseline serum collection, 25/38 (66%) patients in the AHSCT group were receiving DMTs, either second-line (*n* = 22) or first-line ones (*n* = 3); the remaining cases were off-treatment and had discontinued DMTs a median of 5.5 months (range 1–42) before transplant. In the SP-MS CTRL group, 13/22 (59%) patients were receiving DMTs at the time of sample collection (a second-line treatment in nine cases), while the remaining nine patients had been off-treatment for at least 6 months (median 11.5 months, range 7–84; data not shown).

### sNfL Concentration Analysis

#### sNfL Concentration in the Control Groups

**Figure 1:**
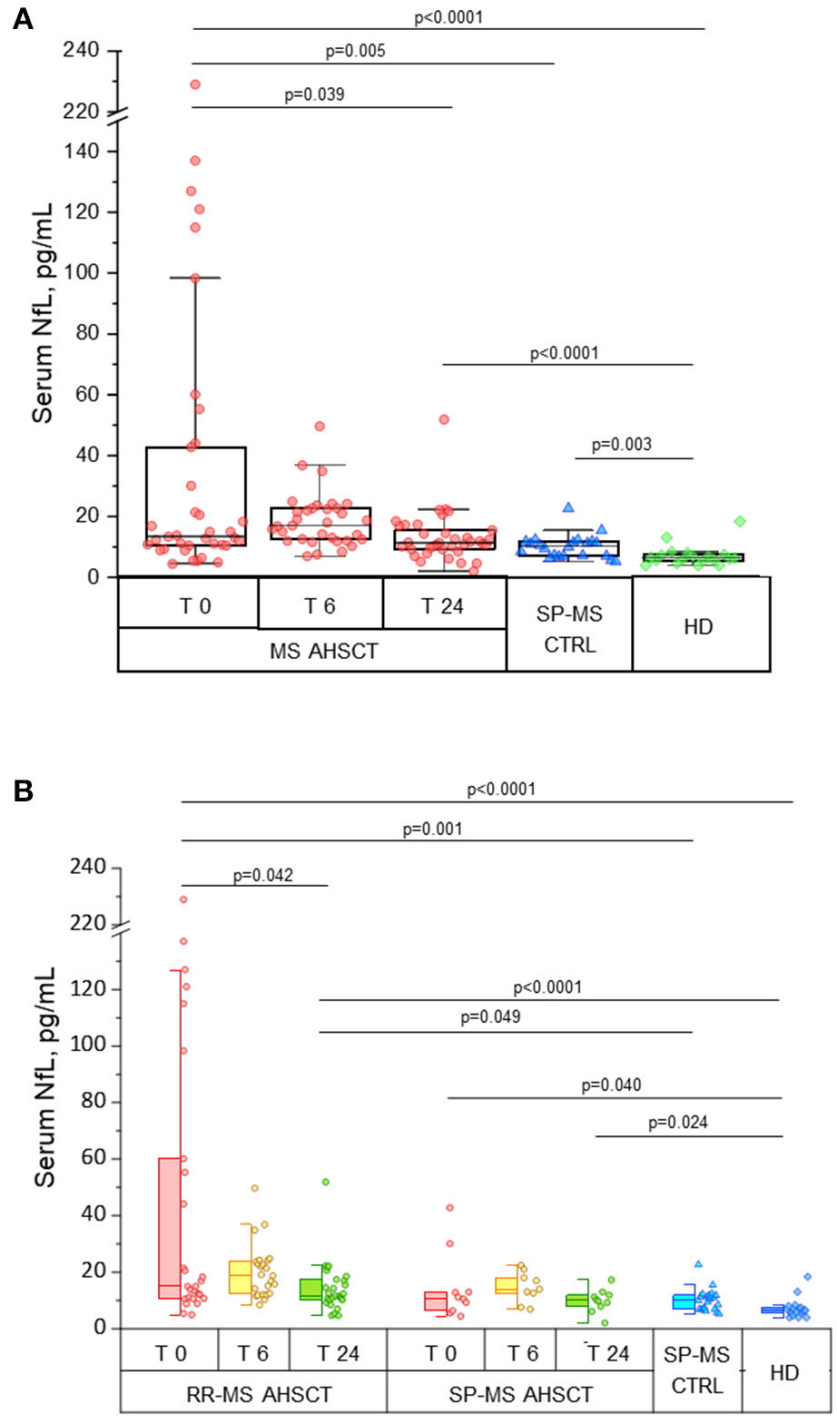
Intermediate-intensity autologous haematopoietic stem cell transplantation reduces serum neurofilament light chain concentrations in treated MS patients. Serum neurofilament light chain (sNfL) concentrations in patients affected by aggressive relapsing-remitting (RR-) or secondary-progressive (SP-) multiple sclerosis (MS) before (T 0) and at months 6 (T 6) and 24 (T 24) following autologous haematopoietic stem cell transplantation (AHSCT, *n* = 38), compared with inactive SP-MS patients (i.e. without signs of recent clinical or radiological disease activity, SP-MS CTRL, *n* = 22) and with healthy individuals (HD, *n* = 19). **(A)** Overall MS AHSCT cohort. In AHSCT patients, baseline values of sNfL (median 13.4 pg/mL, range 4.4–229) were higher than in both SP-MS CTRL and HD groups (median 10.25 pg/mL, range 5.2–22.6, and median 6.4 pg/mL, range 4.0–18.4, *p* = 0.005 and <0.0001, respectively). SNfL at month 6 after transplant did not change compared to baseline (*p* = 0.427), but at T24 a reduction compared to baseline was observed (p=0.039), reaching levels similar to SP-MS CTRL (*p* = 0.110) but being still higher than in HD (*p* < 0.0001). **(B)** RR-MS and SP-MS AHSCT subgroups. At baseline, sNfL concentration in RR-MS cases was higher than in SP-MS CTRL (*p* = 0.001) and HD groups (*p* < 0.0001), whereas SP-MS AHSCT cases showed sNfL levels different from HD only (*p* = 0.040). SNfL concentrations at month 24 were reduced compared to baseline in the RR-MS AHSCT group (*n* = 28; 11.7 pg/mL, range 4.6–51.9, and 15 pg/mL, range 4.9–229, respectively; *p* = 0.042), whereas they did not differ from baseline in the SP-MS AHSCT group (*n* = 10; 10 pg/mL, range 2.0–17.3, and 10.8 pg/mL, range 4.4–42.8, respectively; *p* = 0.721). sNfL at month 24 were higher in RR-MS AHSCT cases compared both to SP-MS CTRL (*p* = 0.049) and HD (*p* < 0.0001); in SP-MS AHSCT group, values were higher than HD (*p* = 0.024).

Median sNfL concentration was higher in the SP-MS CTRL group than the HD group, being 10.25 (5.2–22.6) pg/mL and 6.4 (4.0–18.4) pg/mL, respectively, *p* = 0.003 (Figure 1). No differences were observed between SP-MS CTRL patients who were receiving DMTs at the time of blood sampling and those who were not: 9.39 (range 5.2–12.4) pg/mL and 11.2 (range 7–22.7) pg/mL, respectively (*p* = 0.235; data not shown). A moderate correlation between sNfL concentrations and age at sample was observed in both groups, with r=0.56 in SP-MS CTRL (*p* = 0.007) and *r* = 0.46 in HD (*p* = 0.045; data not shown).

#### Baseline sNfL Concentrations in the MS AHSCT Group

In AHSCT patients, median sNfL concentration at baseline was 13.4 (range 4.4–229) pg/mL (Figure 1A), being 15 (range 4.9–229) pg/mL in RR-MS and 10.8 (range 4.4–42.8) pg/mL in SP-MS cases (Figure 1B). Median sNfL at baseline were higher in the MS AHSCT group compared to the SP-MS CTRL and HD groups, both considering the whole AHSCT cohort (*p* value 0.005 and < 0.0001, respectively; Figure 1A) and the RR-MS AHSCT subgroup (*p* = 0.001 and < 0.0001, respectively; Figure 1B). In the SP-MS AHSCT subgroup, median baseline sNfL concentration was similar to that of the SP-MS CTRL group (*p* = 0.665; Figure 1B) but it was higher than in HD (*p* = 0.040).

No differences in baseline sNfL were observed between MS AHSCT patients who were receiving DMTs at the time of baseline sample collection and those who were not (13.6 pg/mL, range 4.9–137, and 12.4 pg/mL, range 4.4–229, respectively, *p* = 0.404; data not shown). sNfL concentrations at baseline correlated with clinical and/or MRI markers of recent inflammatory disease activity (Table 2).

**Table 2:** Correlation between serum neurofilament light chain levels at baseline and clinical-radiological characteristics of the MS AHSCT patients corrected for age at sampling.

	**R**	***p*** **value**
Relapses previous year, n	0.53	0.001
Days since last relapse	−0.40	0.030
Relapse in the previous 6 months, yes	0.38	0.026
Gd+ lesions, *n*	0.48	0.003
Gd+ lesions, volume	0.66	<0.001
Delta-EDSS in the previous year	0.37	0.029
EDSS worsening in the previous year, yes	0.46	0.006
T2 lesion load at baseline, mm^3^	0.54	0.004

#### sNfL Variation Following AHSCT

**Figure 2:**
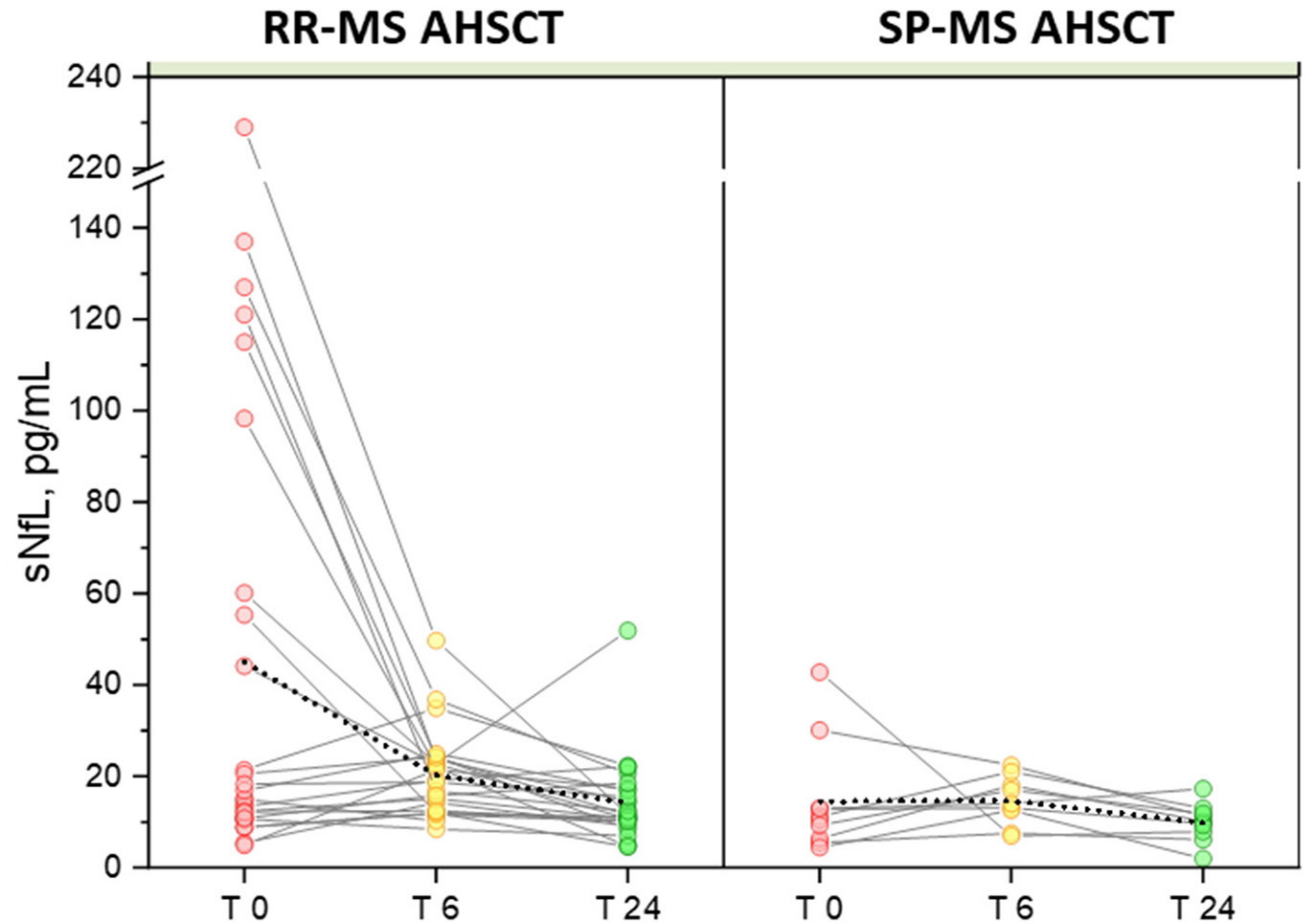
Variation in serum NfL following AHSCT. Individual values of pre- and post-AHSCT sNfL for RR-MS **(left)** and SP-MS AHSCT patients **(right)** are connected with solid colour lines. Mean values of each group are connected with a dotted line, showing high sensitivity of the mean to the outliers with a significant reduction in the RR-MS group at months 6 (20.23 pg/mL) and 24 of follow-up (14.33 pg/mL) compared to baseline (45.06 pg/mL), *p* values 0.012 and 0.010 for month 6 and 24, respectively.

In the MS AHSCT group, median sNfL concentration at month 24 (11.3 pg/mL, range 2.0–51.9) was remarkably reduced compared to baseline (*p* = 0.039), whereas levels at month 6 (17 pg/mL, range 6.9–49.7) did not differ from baseline (*p* = 0.427; Figure 1A). At month 24, median sNfL concentration in the MS ASHCT group was similar to that of the SP-MS CTRL group (p=0.110), but it was still higher than in HD (*p* < 0.0001; Figure 1A). Variation of individual values of MS AHSCT patients are reported in Figure 2. A median decrease by 13% (median 42.8 pg/mL, range 2.4–217.3) of the baseline value was observed, which was by at least 50% in 13 patients (10 RR-MS, 3 SP-MS), reaching up to 90% in four cases.

In the RR-MS AHSCT subgroup, median sNfL concentrations were reduced at month 24 (11.7 pg/mL, range 4.6–51.9) compared to baseline (15 pg/mL, range 4.9–229; *p* = 0.042) (Figure 1B).

In the SP-MS AHSCT subgroup, sNfL levels at months 24 (10 pg/mL, range 2.0–17.3) were similar to baseline (10.8 pg/mL, range 4.4–42.8; *p* = 0.721) (Figure 1B).

A remarkable elevation in sNfL at month 24 (51.9 pg/mL) compared to month 6 (22.7 pg/mL) was observed in one single case (RR-MS) who had experienced a clinical and radiological disease reactivation (i.e. relapse associated with occurrence of new Gd+ lesions at brain MRI) 2 months before the blood sample collection. This patient had received BEAM without melphalan and ATG for safety issues.

Baseline sNfL concentrations correlated with those at month 6 (*r* = 0.56, *p* = 0.001), but not with 24-months values (*p* = 0.547; data not shown).

### Relapses and Disability Following AHSCT

**Figure 3:**
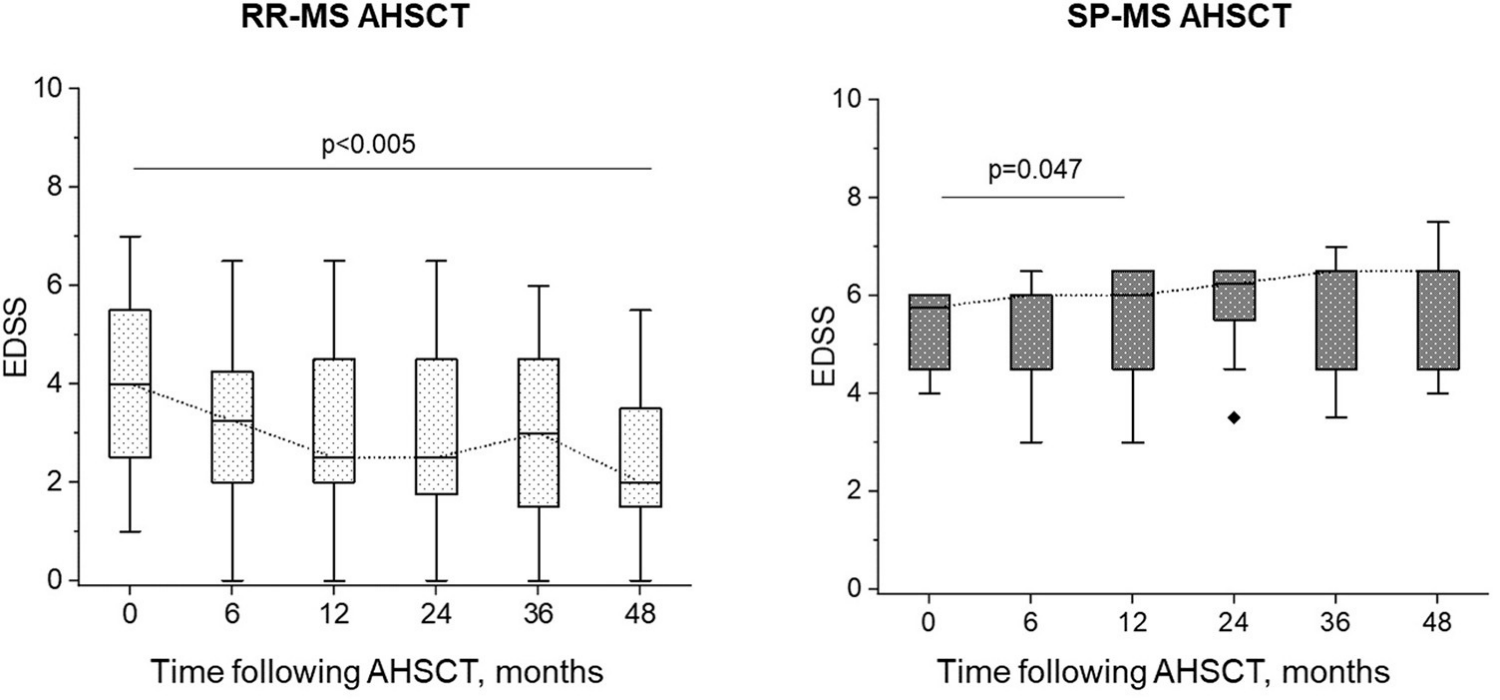
EDSS change following AHSCT. In the RR-MS AHSCT subgroup, EDSS decreased compared to baseline at each timepoint (*p* <0.005), whereas in the SP-MS AHSCT subgroup, median EDSS increased compared to baseline at month 24 after AHSCT (*p* = 0.047).

ARR dropped from 1.13 in the two years before AHSCT to 0.0054 up to the last follow-up after transplant (median 49 months, range 24–153). All the cases except for one were relapse-free up to the last follow-up, being relapse-free survival from year 2 to last follow-up 97%. At month 24, EDSS worsening was observed in 8/38 cases (21%, 1/28 RR-MS−4%, and 7/10 SP-MS−70%, *p* < 0.0001), occurring at a median of 10 months (2–24) after the procedure. One additional worsening was reported in one SP-MS case at month 66. Four aMS patients (all SP-MS) experienced CDA and the second confirmed episode of progression was reported at a median of 27 months (range 25–37) of follow-up. In the RR-MS subgroup, EDSS improved at last follow-up in 13/28 (46%) cases, stabilised 14/28 (50%) and worsened in 1 case (4%). Median EDSS in the RR-MS AHSCT subgroup improved at all the timepoints following AHSCT compared to baseline (*p* < 0.005, Figure 3), with a confirmed decrease up to −4.0 EDSS points. Following AHSCT, a slight deterioration of disability was observed in the SP-MS subgroup, which was significant at month 24 compared to baseline (*p* = 0.047; Figure 3), being the median worsening of 1.0 EDSS point (range 0.5–1.5).

### Safety

No fatalities or life-threatening complications were observed; common adverse events following transplant were aligned with the literature (20).

### MRI Activity and Brain Atrophy

One case (RR-MS) showed new Gd+ lesions at month 22, associated with clinical relapse. All the remaining cases were free from both new and Gd+ lesions up to the last follow-up.

**Figure 4:**
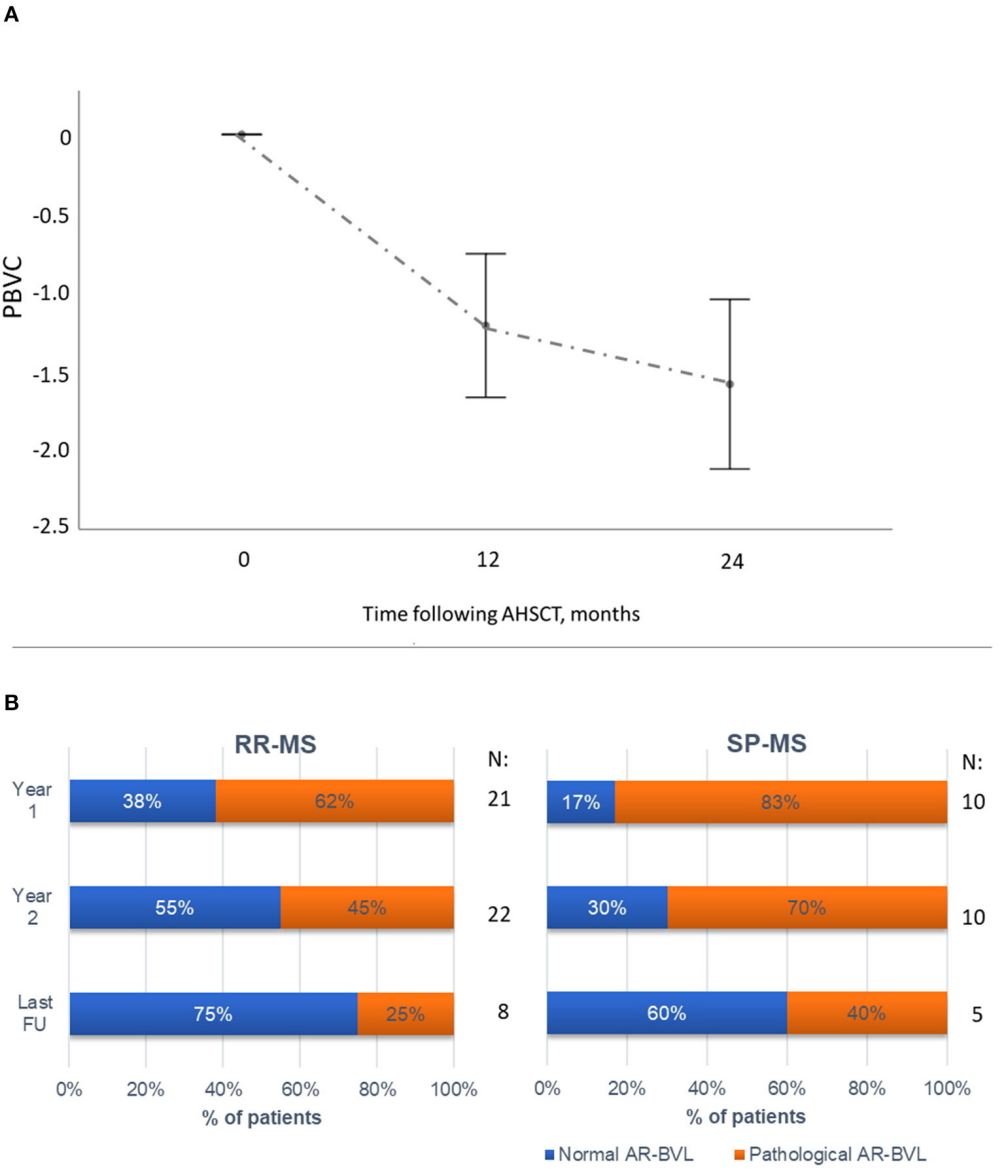
Brain atrophy following AHSCT. **(A)** Mean (95% CI) percentage of brain volume change (PBVC) in the first two years following AHSCT in the RR-MS and SP-MS AHSCT cases, compared to the baseline scan. The greatest reduction in PBVC was observed during the first year following AHSCT, followed by a reduction in AR-BVL. **(B)** Annualised rate of brain volume loss (AR-BVL) in RR- and SP-MS AHSCT cases at year 1, 2 and up to last follow-up available beyond year 2. The proportion of patients with normalisation of AR-BVL tended to be higher in RR vs. SP-MS cases and increased over follow-up.

Brain volume loss after transplant was evaluated in 32 patients (22 RR-MS and 10 SP-MS) who had scans of adequate quality to perform the analysis. Mean PBVC was −1.15 (95%CI −1.55, −0.75) and −1.56 (95%CI −2.04, −1.09) at months 12 and 24, respectively (Figure 4A). MRI scan at month 6 following AHSCT was available for 18 cases (14 RR-MS, 4 SP-MS), and mean PBVC at this timepoint was −0.6 (95%CI −1.02, −0.17). At year 2 following AHSCT, 15/32 cases (47%) showed normalisation of AR-BVL, without re-baseline (median AR-BVL −0.24%, range −0.4–0.0); at this timepoint, the observed AR-BVL was within normal values for age (i.e. below−0.4%) in 55% of the RR-MS AHSCT and 30% of the SP-MS AHSCT cases analysed (*p* = 0.197, Figure 4B). The normalisation of AR-BVL did not correlate with EDSS worsening following transplant (data not shown). AR-BVL up to the last follow-up beyond year 2 (median 7 years, range 3–12) was available for eight RR-MS and five SP-MS cases and it normalised in 75% and 60% of the cases, respectively, *p* = 0.207 (Figure 4B).

### Association Between sNfL Concentration and Outcomes

No correlations between sNfL concentrations either at baseline or follow-up and disability accrual or AR-BVL normalisation were observed. In the SP-MS AHSCT subgroup, baseline sNfL concentration did not predict subsequent disability accrual up to the last follow-up (HR 1.02, 95%CI 0.97–1.08, *p* = 0.442), nor did month 6 sNfL levels (HR 1.12, 95%CI 0.90–1.40, *p* = 0.306). sNfL concentration at month 24 did not differ between patients who had shown disability accrual within 24 months from AHSCT and those who had not (*p* = 0.299 for the MS AHSCT and 0.667 for SP-MS AHSCT groups; data not shown).

sNfL at month 24 did not correlated with T2 lesion load at the same timepoint (R −0.24, *p* = 0.187; data not shown).

## Discussion

sNfL concentrations were evaluated in 38 aMS patients who underwent intermediate-intensity AHSCT at our centre, and the results were compared with those detected in two control groups: SP-MS cases without recent inflammatory activity (i.e. relapses or new/Gd+ lesions in the previous 6 months) and individuals not affected by neurological diseases (HD). The main aim of the study was to assess the impact of intermediate-intensity AHSCT on sNfL in RR-MS and SP-MS patients undergoing the procedure, exploring whether transplant could induce a reduction in sNfL to levels similar to the control groups. The potential role of sNfL as a marker of disability accrual independent from new focal inflammation was also investigated in AHSCT treated MS patients.

Patients enrolled in the transplant program showed highly active disease, refractory to DMTs in most of the cases. The procedure was effective in halting relapses and new inflammatory MRI activity in all the patients except for one RR-MS, who relapsed at month 22, after being treated with BEAM without melphalan and ATG for safety issues.

SP-MS AHSCT patients had a more aggressive disease course than SP-MS CTRL, as expected for a selection bias due to the enrolment in the AHSCT program. Treatment with DMTs did not influence sNfL concentrations in our sample, both in the SP-MS CTRL and MS AHSCT groups, and two opposite explanations can be offered: the lack of recent inflammatory activity in all the cases in the SP-MS CTRL group and the occurrence of breakthrough disease activity despite treatment in the MS AHSCT group. Superimposed inflammatory activity which could overcome the small age-related increase in sNfL concentration might explain the lack of correlation between sNfL and age at sampling in the MS AHSCT group only (21).

Aligned with previous works (5), sNfL reflected recent inflammatory activity: values were higher in RR-MS compared to SP-MS patients, consistent with the differences in recent relapses and MRI activity between the groups. The transient although not significant increase in median sNfL concentration detected at month 6 after AHSCT might mirror transient neuronal damage with consequent release of NfL in the CSF and serum. However, the interpretation of these data is not univocal and two not mutually exclusive scenarios can be hypothesised: a transient neuro-toxic effect of the chemotherapy, or accelerated neuronal damage induced by a rapid suppression of inflammation. Potential neurotoxicity of AHSCT in MS patients was suggested by previous studies, which however were limited to high-intensity conditioning regimens only. An increase in serum neurofilament heavy chains was reported in SP-MS patients treated with cyclophosphamide and total body irradiation, a protocol no longer used due to safety concerns (10). More recently, a transient increase in sNfL was observed 3 months after busulfan-cyclophosphamide AHSCT, followed by a decrease to levels similar to baseline starting in month 6 (8). Albeit valuable, caution should be adopted in inferring these observations to intermediate- or low-intensity regimen AHSCT, and no evidence of neurotoxicity of these protocols is available so far. Furthermore, the observed increase in sNfL might be due to an MS-specific process rather than to a neurotoxic effect of the chemotherapy, i.e. the consolidation of baseline axonal damage in a CNS affected by inflammation could be the main driver of NfL release. If this latter hypothesis was true, the transient increase in sNfL observed could be an epiphenomenon of the glial scarring of MS lesions “de-activated” by the procedure. Potentially supporting this hypothesis, a transient increase in sNfL was reported in a proportion of MS patients receiving alemtuzumab at months 2–3 following either the first or second course of treatment (5/15 patients and 3/15 cases, respectively) (22). Finally, in either case, indirect proof of the ability of the drugs adopted during mobilisation and/or conditioning to cross the blood-brain barrier might be provided, thus suggesting that AHSCT might be effective also on compartmentalised inflammation. If this latter hypothesis is true, AHSCT could become a suitable treatment option for patients in progressive phases of MS with signs of ongoing compartmentalised inflammation, where the lack of effective approved DMTs is still an unmet clinical need.

Furthermore, potential pleiotropic effects of AHSCT have been suggested, such as a possible contribution to tissue repair mediated by putative trans differentiation of the graft in neural/glia cells and trophic/protective effects on CNS tissue, although further research is needed to explore this issue (23, 24).

The significant decrease of sNfL detected at month 24 compared to baseline confirms that the procedure exerts a long-standing effect in reducing inflammation-related axonal damage, at least in the RR-MS form, where the greatest reduction was observed. The amount of decrease in sNfL levels detected is similar to that previously reported in a cohort of patients treated with high-intensity AHSCT (7), suggesting that BEAM-AHSCT might be as effective as the former protocol in suppressing inflammation-related axonal damage. Notably, the great reduction in sNfL after transplant is remarkable considering that most of the MS AHSCT cases in the present cohort were receiving DMTs at the time of baseline sample collection, providing further evidence on AHSCT as an effective escalating therapy.

sNfL concentrations at month 24 did not differ between MS AHSCT cases and SP-MS CTRL, thus reflecting the resolution of new inflammatory activity in all the cases, except for one patient who experienced a disease reactivation. Indeed, this latter was the only case that showed a marked increase at month 24 compared to month 6.

Correlation between sNfL levels and disability accrual and brain atrophy has been reported in large cohorts of non-AHSCT treated MS patients (4, 25), whereas in the present study baseline sNfL were not able to predict response to AHSCT in terms of disability progression or brain atrophy, and sNfL reflected mainly new focal inflammatory activity. Although the small sample size could have prevented us from finding significant correlations, it could be speculated that this difference might be due, at least in part, to a different persistence of new focal inflammatory activity in untreated or various DMTs treated patients described in other studies (4, 25) and AHSCT-treated patients, being new focal inflammation virtually suppressed in these latter. It could be hypothesised that sNfL concentrations might correlate with prognosis in MS as long as new inflammation (relapses and/or new MRI lesions) is the main driver of disability accrual. Following AHSCT, the persistence of median values of sNfL higher than in HD could be explained by a background of axonal damage persisting in a subset of treated patients despite the suppression of new inflammatory activity, and possibly due to non-inflammation driven neurodegeneration or by compartmentalised inflammation not eradicated by AHSCT. However, the lack of correlation with clinical outcomes partially argues against this hypothesis, even if the study might be underpowered for this purpose. Moreover, the measurement of NfL in the serum, where values are considerably lower than in CSF, might have a low sensitivity to detect pathological release of small amounts of NfL promoted by neurodegeneration or smouldering inflammation, suggesting that sNfL might not be a sensitive surrogate marker of these latter phenomena in progressive disease.

In this study, the effectiveness of AHSCT on ARR and MRI activity was aligned with the literature (26–28). A significant improvement in median EDSS was observed in the RR-MS AHSCT subgroup, while disability progression occurred exclusively in SP-MS cases. AR-BVL was high during the first year following treatment and this could be due, at least in part, to pseudoatrophy, i.e. shrinking of brain tissue due to rapid resolution of inflammation and of the associated oedema. A normalisation of AR-BVL two years after transplant was observed in 15 out of 32 evaluable cases (47%), mostly RR-MS (12/15, 80%). The safety of the procedure was overall acceptable.

Our study has several limitations. First of all, the lack of a control group of active MS patients not undergoing AHSCT does not allow to compare the relative effect of AHSCT on sNfL; moreover, as MS AHSCT patients showed highly active disease before the enrolment, a regression to the mean could, at least in part, influence the reduction in sNfL observed shortly after transplant. Despite broad experience in AHSCT for MS in our centre, the relatively small sample size could have prevented us from identifying significant correlations between sNfL and the outcomes. Furthermore, the lack of blood samples collected shortly after transplant could have underestimated a potential increase in sNfL occurring early after the procedure, as already pointed out by other authors (8), therefore no conclusive data on a potential neuro-toxicity of intermediate-intensity AHSCT in MS could be provided.

## Conclusion

The present study provides class IV evidence on the efficacy of intermediate intensity AHSCT in inducing suppression of new inflammatory activity in aggressive MS, both on clinical and para-clinical parameters. The remarkable reduction in sNfL observed in aMS patients who were receiving DMTs at baseline strengthens the role of AHSCT as an effective escalating therapy, providing that the switch to transplant is performed timely before the occurrence of irreversible disability accrual. Further data are needed to properly answer the question as to whether an early (although not significant) increase in sNfL after AHSCT might harbour a neurotoxic effect of the procedure vs. consolidation of pre-existing axonal damage induced by a rapid suppression of inflammation.

In our sample, sNfL did not perform as a sensitive surrogate marker of inflammation-independent neurodegeneration but was reliably associated with recent focal inflammatory activity, and baseline levels could not predict response to treatment in terms of disability accrual or brain atrophy. The individuation of a biomarker that could identify MS patients in whom axonal damage is still related to inflammation (and could therefore be eradicated by maximal immunosuppression) is of pivotal importance to allow a better selection of the patients who might benefit from anti-inflammatory treatments, including transplant.

## Data Availability Statement

The raw data supporting the conclusions of this article will be made available by the authors, without undue reservation.

## Ethics Statement

The studies involving human participants were reviewed and approved by Comitato Etico Area Vasta Centro, University Hospital of Careggi. The patients/participants provided their written informed consent to participate in this study.

## Author Contributions

AM contributed to the conception and design of the study, acquired and analysed data, and wrote the first draft of the manuscript. LeM, SF, and MP performed imaging analyses and acquired related data. CI, AB, CM, and RS acquired clinical data. MD contributed to the database. FM, BM, and TB acquired laboratoristic data. LuM and AR contributed to the conception and design of the study and reviewed the manuscript. All authors contributed to manuscript revision, read, and approved the submitted version.

## Conflict of Interest

The authors declare that the research was conducted in the absence of any commercial or financial relationships that could be construed as a potential conflict of interest.

## Publisher's Note

All claims expressed in this article are solely those of the authors and do not necessarily represent those of their affiliated organizations, or those of the publisher, the editors and the reviewers. Any product that may be evaluated in this article, or claim that may be made by its manufacturer, is not guaranteed or endorsed by the publisher.
